# Schizotypal Traits are Associated with Poorer Executive Functioning in Healthy Adults

**DOI:** 10.3389/fpsyt.2015.00079

**Published:** 2015-06-01

**Authors:** Stephanie Louise, Caroline Gurvich, Erica Neill, Eric J. Tan, Tamsyn E. Van Rheenen, Susan Rossell

**Affiliations:** ^1^Monash Alfred Psychiatry Research Centre (MAPrc), Alfred Hospital, Central Clinical School, Monash University, Melbourne, VIC, Australia; ^2^Faculty of Health, Arts and Design, Brain and Psychological Sciences Research Centre, Swinburne University of Technology, Melbourne, VIC, Australia

**Keywords:** schizotypy, schizotypal traits, psychosis proneness, cognition, executive functioning, attention, inhibition, memory

## Abstract

Previous research has shown mild forms of the neurocognitive impairments seen in schizophrenia among healthy individuals exhibiting high schizotypal traits. This study aimed to explore associations between schizotypy and cognitive performance in an adult community sample. Ninety-five females and 79 males completed the Oxford–Liverpool Inventory of Feelings and Experiences (O-LIFE), which measures four separable aspects of schizotypy: cognitive disorganization, unusual experiences, introvertive anhedonia, and impulsive non-conformity. Subsequently, participants were administered a neurocognitive battery incorporating measures of executive skills including inhibition, cognitive flexibility, reasoning, and problem solving along with measures of attention and processing speed and both verbal and spatial working memory. In line with predictions, the current study found that higher scores on the subscales of unusual experiences, cognitive disorganization, and impulsive non-conformity related to worse performance on a measure of inhibition. Additionally, as introvertive anhedonia increased, both attention and processing speed and reasoning and problem-solving performance became more impaired. In conclusion, this study extends schizotypy literature by examining the subscales of the O-LIFE, and enables inferences to be drawn in relation to cognitive impairment in schizophrenia.

## Introduction

Schizophrenia generally a lifelong psychiatric illness associated with distressing mood, cognitive, and functional symptoms (1). Cognitive impairment is a key component of schizophrenia and is generally resistant to current treatment medications (2). In addition to an overall decrease in IQ, a range of cognitive impairments have been found to be associated with the disorder, particularly in the areas of “executive functioning,” an umbrella term referring to a range of functions that include the capacity to plan, organize, attend to, monitor, and inhibit behaviors, as well as in the areas of language and memory (3). Such cognitive deficits are likely to be premorbid, that is, they precede the onset of the illness (2, 3). Additionally, they are usually stable or enduring throughout the course of the illness and often remain during symptom remission (2, 3). Cognitive impairments hinder day-to-day functioning and are one of the strongest predictors of clinical, social, and functional outcomes, even more so than positive and negative symptomatology (2, 3). Cognitive functioning is also important to treatment decision-making and can be a good predictor of treatment effects (2).

Mild forms of the cognitive deficits observed in schizophrenia are also found in unaffected first-degree relatives and healthy individuals exhibiting schizotypal traits (4, 5). This has led recent studies to suggest several cognitive measures as potential “endophenotype candidates” or biological markers for the illness (3–5). Schizotypy is a psychological construct involving personality characteristics and perceptions, beliefs, and experiences that are phenomenologically similar to, but less severe than the symptoms of schizophrenia (6, 7). Consequently, schizotypy represents a major focus area of research on schizophrenia and the dimensional approach to it (8). By studying schizotypy in the general population, predisposing and potentially protective factors for schizophrenia can be explored, without the potential confounds of symptoms, motivation deficits, illness chronicity, and treatment medications (9). Recent literature has suggested that schizotypy can be broken down into four factors, which reflect those symptom factors seen in schizophrenia (10).

Positive schizotypy taps into perceptual aberrations, magical thinking, unusual experiences, and hallucinations, and is thought to resemble positive symptomatology in schizophrenia (7, 8). Negative schizotypy encompasses introvertive anhedonia, in particular, a lack of social and physical enjoyment and an avoidance of social connections, which is suggested to reflect negative symptomatology (7, 8). Cognitive disorganization taps into deficits in decision-making abilities, concentration, attention, language, and thought disorder (7, 8). Lastly, asocial behavior taps into impulsive non-conformity, such as reckless, harmful, or disinhibited behaviors (7).

Poorer neurocognitive performance similar to that seen in schizophrenia, albeit in a milder form, has been identified in individuals exhibiting high levels of schizotypy traits (11). For example, inferior levels of attention and executive functioning have been revealed: Cimino and Haywood (12) found healthy individuals high in schizotypy traits, based on a mean of all Oxford–Liverpool Inventory of Feelings and Experiences (O-LIFE; a self-report inventory assessing schizotypy) factor scores, to exhibit significantly more errors and longer latencies on the Stroop Color–Word Interference Test, in comparison with individuals low in schizotypal traits. This finding is indicative of relative impairments in inhibition and attentional switching or cognitive flexibility (12). However, findings of cognitive disinhibition in schizotypy have not always been consistent. For example, some studies have found no significant associations between schizotypy factors and Stroop Color–Word Interference Test performance (13, 14). Conversely, using the Wisconsin Card Sorting Test (WCST), both Gooding et al. (11) and Kim et al. (15) revealed comparative deficits in cognitive flexibility in university students exhibiting high levels of schizotypal traits. This was evidenced by fewer categories achieved and increased preservative errors compared with controls (11, 15).

Additionally, further evidence for poorer performance in attention and executive functioning has been identified by past research. Using the O-LIFE in a university sample, Rawlings and Goldberg (16) revealed a significant association between positive schizotypy and decreased sustained attention, as evidenced by a positive relationship between the cognitive disorganization factor of the O-LIFE and poorer performance on the Continuous Performance Task. Chen et al. (9) had similar findings, however, also found an association between negative schizotypy and poor performance on the Continuous Performance Task.

Relative deficits in verbal and spatial working memory have also been identified by previous research. For instance, using a university sample, Matheson and Langdon (17) found that once age was controlled for verbal working memory, as evidenced by correct manipulations on the Letter–Number Sequencing Task, was a significant predictor of cognitive, perceptual, and negative interpersonal schizotypal traits. Similar associations between schizotypy and spatial working memory have also been revealed by previous studies (18). However, findings between working memory and schizotypy have not always been replicated, for example, Lenzenweger and Gold (19) did not identify a significant relationship between positive schizotypy and verbal working memory (Letter–Number Sequencing Task).

Only a small number of studies have examined cognitive functioning in relation to separate schizotypy factors. These studies suggest that lowered performance in attention, executive functioning, and sustained attention (using a Continuous Performance Task) is related to higher scores on the cognitive disorganization schizotypy factor as well as negative schizotypy (9, 16).

Taken together, these findings suggest that high schizotypy is associated with reduced cognitive ability (albeit milder than that seen in schizophrenia). However, this is a very broad finding and more fine-grained analysis of the nature of this relationship is required. As discussed, there are currently only a small number of articles examining the schizotypy subtypes (9, 16). Furthermore, past schizotypy research has predominantly relied upon adolescent or university educated samples who are unlikely to be a good match to the schizophrenia population generally. By using a sample of adults over the average age for schizophrenia onset, it is assumed that the schizotypal traits exhibited by individuals are likely to lie within healthy limits and therefore are not dormant symptoms of psychopathology (20). Additionally, previous literature has identified first-degree relatives of patients with schizophrenia to score significantly higher and with more variation for both positive and negative schizotypy compared with controls (21). Consequentially, by using extensive exclusion criteria that restricts the presence of individuals with a current psychiatric illness or with a family history of a schizophrenia spectrum disorder or bipolar disorder, the dormant symptoms of psychopathology are further controlled for and the sample is therefore likely to be more homogenous in regards to schizotypal traits. Lastly, due to primarily small sample sizes in previous studies, the need for the exploration of the association between schizotypy and cognition and a large sample is evident.

Based on shortcomings in the literature, the current study aimed to explore the relationship between the four-schizotypy factors defined in the O-LIFE and those areas of cognition, which have previously been found to relate to schizotypy (inhibition, cognitive flexibility, attention, processing speed, and reasoning and problem solving) using traditional neurocognitive tasks. This study will address previous limitations by (a) looking at the relationship between individual schizotypy factors of the O-LIFE and cognition, (b) using a large sample, (c) over the typical age for schizophrenia onset, and (d) free from genetic liability and current psychopathology.

Given previous findings of inhibitory deficits in schizophrenia patients and high schizotypy samples, it was hypothesized that higher scores on the unusual experiences and cognitive disorganization factors of the O-LIFE would relate to poorer inhibition and cognitive flexibility, as measured by the Color–Word Interference Test performance. Additionally, in line with previous schizotypy literature, it was predicted that the introvertive anhedonia factor of the O-LIFE would negatively associate with reduced attention and processing speed, as measured by the Trail Making Test – Part A. Furthermore, it was hypothesized that there would be a negative association between the unusual experiences, introvertive anhedonia and impulsive non-conformity factors, and reasoning and problem-solving performance, as measured by a Mazes task. Lastly, based on previous findings in working memory and schizotypy, a negative relationship was also expected between both the unusual experiences and introvertive anhedonia factors, and verbal and spatial working memory performance, as measured by the Letter–Number Sequencing and Spatial Span Tasks. Furthermore, exploratory analyses of the four-schizotypy factors and all neurocognitive variables will be conducted.

## Materials and Methods

### Participants

Potential participants voluntarily responded to advertising through flyers at local community centers and the researcher’s private social media pages. Following telephone screening, 175 healthy adults between 18 and 64 years of age (95 women and 79 men) met participation inclusion criteria. Participants were excluded from the study if they had a current psychiatric illness; history of or first-degree biological relative with schizophrenia or schizoaffective disorder; current use of a psychotropic drug; or, history of substance abuse or neurological illness. Demographic information revealed that one included participant was adopted (hence, knowledge of their biological relatives’ psychiatric history was unknown), and one participant was color blind. The participant who was color blind was excluded from all color–word interference tasks. All participants were financially reimbursed for their time and travel costs. The Alfred Health Human Ethics Committee and the Monash University Standing Committee on Ethics in Research in Humans approved all experimental procedures and informed written consent was obtained by all participants in accordance with these ethical requirements.

### Materials

#### Screening Interview

*The Mini-International Neuropsychiatric Interview (M.I.N.I.)*, a clinician-rated brief structured psychiatric interview compatible with DSM-IV diagnostic criteria, was used to screen for the presence of psychiatric conditions (22).

*The Montgomery Äsberg Depression Rating Scale (MADRS)*, a 10-item clinician-rated scale, was used to assess the presence and severity of depressive symptoms, relating to the previous week (23). Items are scored on a six-point Likert scale (0–5) and summed to calculate a total score ranging from 0 to 50, with higher scores indicating greater depression severity (24).

*The Wechsler Test of Adult Reading (WTAR)* included to provide an estimate of intellectual functioning and premorbid verbal intelligence (25).

#### Schizotypy Assessment

*The Oxford–Liverpool Inventory of Feelings and Experiences (O-LIFE)* is a self-report questionnaire designed to measure psychosis-proneness, principally schizotypy in healthy individuals (8). The questionnaire consists of four scales: unusual experiences comprise 30-items and reflect positive symptomatology, for example, “Do you believe in telepathy?” Cognitive disorganization consists of 24-items and reflects cognitive deficits and thought disorder, for example, “Is it hard for you to make decisions?” Introvertive anhedonia comprises 27-items and reflects negative symptomatology, for example, “Do you prefer watching television or going out with people?” Lastly, impulsive non-conformity reflects a-social behavior and consists of 23-items, for example, “Do you at times have an urge to do something harmful or shocking?”

#### Inhibition and Cognitive Flexibility Assessment

*The Delis–Kaplan Executive Function System (D-KEFS), Color–Word Interference Test*, assesses inhibition and switching or cognitive flexibility (25, 26). The test consists of four conditions, each comprising 40-items: condition 1 requires respondents to name patches of color. In the second condition, respondents are required to read color names written in black ink. The third condition requires respondents to name the dissonant ink color that words are written in. In the fourth condition, respondents are required to switch between naming the dissonant ink color and reading the words. Each condition is timed and both self-corrected and unknown errors are summed for each condition to calculate a score for both raw time and total errors, ranging from 1 to 40, with higher scores indicating greater number of errors (25, 26).

For this study, the four variables of interest were: Inhibition V Color Naming raw time, which was a measure of inhibitory latency, once baseline color naming was controlled for by subtracting the raw seconds required for the first condition from the third condition; Inhibition/Switching V Inhibition raw time that was a score of cognitive flexibility or attentional switching, after inhibition was controlled for by subtracting the seconds score of the third condition from the fourth condition; Inhibition/Switching V Color Naming raw time, which was a measure of inhibitory latency and cognitive flexibility, once color naming was controlled for by subtracting the seconds required for the first condition from the fourth condition; and Inhibition/Switching V Word Reading raw time that was a measure of inhibitory latency and cognitive flexibility, after baseline wording reading was controlled for by subtracting the seconds taken for the second condition from the fourth condition.

#### Attention and Processing Speed Assessment

*The Halstead–Reitan Neuropsychological Test Battery (HRB)*, *Trail Making Test – Part A*, was designed to measure attention and processing speed (27). The task requires participants to connect 25 numbers in ascending order that are randomly arranged on a page within an assigned maximum time of 300 s (27). Prior to formal task administration, participants first complete a sample exercise containing eight numbers (27). Administration of the task takes approximately 5 min and the completion time in seconds for the formal component is used as a total score with the number of errors expressed also being recorded (27).

#### Reasoning and Problem Solving Assessment

*The Neuropsychological Assessment Battery (NAB) – Mazes* assesses executive functioning, particularly planning and organization (28). The task requires participants to trace their way through a series of seven mazes of increasing difficulty (28). The time limit for each maze varies with the difficulty level and ranges from 30 to 240 s (28). Mazes are scored based on completion and response speed, with scores ranging from 0 to 26, with higher scores indicating better performance (28).

#### Working Memory Assessment

*The Wechsler Memory Scale – Third Edition (WMS-III) – Letter–Number Sequencing* assesses verbal working memory (29). The task requires the researcher to read a series of numbers and letters and the participant is required to recite the digits back to the researcher, numbers first in ascending order followed by letters in alphabetical order, with the lists increasing in difficulty (29). The task consists of 24 trials and each correct recitation receives a score of one, with individual scores summed to calculate a total score ranging from 0 to 24, with higher scores indicating better working memory performance (29).

*The WMS-III – Spatial Span* assesses spatial working memory and consists of two conditions: forwards and backwards (29). The first condition, forwards, consists of 16 trials and requires the researcher to touch the blocks on the Spatial Span board in an order in which the participants must repeat, with increasing difficulty (29). The second condition, backwards, again consists of 16 trials and participants are required to touch the blocks in the reverse order to that of the researcher, with increasing difficulty (29). Each correct trial obtains a score of 1, with individual scores summed to calculate a total score for each condition, ranging from 0 to 16 (29). Condition scores are then summed to calculate an overall total score ranging from 0 to 32, with higher scores indicating better working memory performance (29).

### Procedure

Following a basic telephone assessment of eligibility, participants completed a demographic questionnaire and the O-LIFE. Subsequently, a brief screening interview took place, consisting of the M.I.N.I. screen and the MADRS. The neurocognitive battery was then administered successively, with counterbalancing used to reduce order effects and fatigue.

## Results

### Data analysis

All raw scores were processed using PASW Version 18 (SPSS Ltd.) to produce the summary data. Although statistical analyses were based on previous literature, due to multiple comparisons, the alpha level for all statistical analyses was set at 0.01, unless otherwise stated.

Prior to analyses, assumption testing was conducted to assess the suitability of the data for a correlation analysis. Following inspection of the Frequency Table, it was found that a small percentage of data was missing from each of the variables (<5% per variable); cases were therefore excluded pairwise for all further statistical analyses. Kolmogorov–Smirnov normality tests revealed that the data violated this assumption and consequently non-parametric tests (Spearman’s rho) were used for all additional analyses.

### Demographics

There were no significant associations between age, years of education, verbal intelligence and depression, as evidenced by the WTAR, MADRS depression scores (see Table 1 for descriptive statistics of the demographic variables; see the Supplementary Material for results of the Spearman’s Rho analyses for neurocognitive variables by schizotypy factors) schizotypy factors scores and the neurocognitive variables (*p* = 0.017–0.908). Consequently, these variables were not controlled for in further analyses.

**Table 1:** **Descriptive statistics for demographic variables**.

	Mean	SD	Min	Max
Age	34.05	13.66	18	64
Years of education	16.27	2.83	9	27
WTAR scaled score	112.30	8.11	83	129
MADRS	1.79	3.01	0	26

*WTAR, Wechsler Test of Adult Reading; MADRS, Montgomery Äsberg Depression Rating Scale*.

### Descriptive statistics and schizotypy factor scores

The descriptive statistics for all neurocognitive variables are presented in Table 2. This table reveals the highest schizotypy factor scores to be for cognitive disorganization and impulsive non-conformity.

**Table 2:** **Descriptive statistics for all neurocognitive variables and schizotypy factors**.

	*N*	Missing *N*%	Mean	SD	Range	TR
O-LIFE
Unusual experiences	171	2.3	4.98	4.86	0–25	0–30
Cognitive disorganization	171	2.3	7.22	5.24	0–20	0–24
Introvertive anhedonia	170	2.9	4.16	3.63	0–20	0–27
Impulsive non-conformity	172	1.7	7.20	4.13	0–19	0–23
D-KEFS Inhibition V Color Naming	168	4	10.54	8.21	−5 to 37	–
D-KEFS Inhibition/Switching V Inhibition	170	2.9	5.88	7.47	−12.4 to 28.20	–
D-KEFS Inhibition/Switching V Color Naming	170	2.9	12.81	11.60	−4 to 49.6	–
D-KEFS Inhibition/Switching V Word Reading	170	2.9	14.89	14.69	−4 to 57	–
Trail Making Test – Part A	173	1.1	25.56	8.91	11–68	300 s
Mazes	172	1.7	10.61	5.65	3–26	0–26
Letter–number sequencing	173	1.1	16.79	2.59	10–24	0–24
Spatial span backwards	175	0	8.95	1.80	2–14	0–16

*Range, observed range of scores; TR, theoretical range of scores; O-LIFE, Oxford–Liverpool Inventory of Feelings and Experiences; D-KEFS, Delis–Kaplan Executive Function System*.

*D-KEFS variables are measured in raw seconds*.

To further explore relationships between schizotypy factors and neurocognitive variables, a two-tailed Spearman’s rho correlation analysis was conducted and results are presented below.

### Inhibition and cognitive flexibility

In terms of assessing inhibition, the analysis revealed a significant positive association between unusual experiences and inhibition versus color naming raw time [*r_s_*(164) = 0.333, *p* = 0.000], inhibition/switching versus color naming raw time [*r_s_*(166) = 0.347, *p* = 0.000], and inhibition/switching versus word reading raw time [*r_s_*(166) = 0.345, *p* = 0.000], accounting for 11.08, 12.04, and 11.90% of shared variance, respectively.

The Spearman’s rho analysis also revealed a significant positive relationship between cognitive disorganization and inhibition versus color naming raw time [*r_s_*(164) = 0.21, *p* = 0.007], inhibition/switching versus color naming raw time [*r_s_*(166) = 0.24, *p* = 0.002], and inhibition/switching versus word reading raw time [*r_s_*(166) = 0.258, *p* = 0.001], accounting for 4.41, 5.76, and 6.66% of shared variance, respectively. The analysis also identified a non-significant positive trend between cognitive disorganization and inhibition/switching versus inhibition [*r_s_*(166) = 0.189, *p* = 0.014].

Additionally, the exploratory analysis showed a significant positive association between impulsive non-conformity and inhibition versus color naming raw time [*r_s_*(165) = 0.450, *p* = 0.000], inhibition/switching versus color naming [*r_s_*(167) = 0.453, *p* = 0.000], and inhibition/switching versus word reading [*r_s_*(167) = 0.433, *p* = 0.000], which accounted for 20.25, 20.52, and 18.75% of shared variance, respectively. Furthermore, a non-significant positive trend was identified between impulsive non-conformity and inhibition/switching versus inhibition [*r_s_*(167) = 0.168, *p* = 0.030].

### Attention and processing speed

In regards to attention and processing speed, the Spearman’s rho analysis showed a significant positive relationship between introvertive anhedonia and Trail Making Test – Part A [*r_s_*(168) = 0.26, *p* = 0.001], accounting for a small amount of shared variance (6.76%).

### Reasoning and problem solving

In respect to planning and organization, a significant negative relationship between introvertive anhedonia and mazes raw score [*r_s_*(168) = −0.212, *p* = 0.006] was revealed by analysis that accounted for a small amount of shared variance (4.49%). Conversely, analysis also showed a significant positive association between impulsive non-conformity and mazes raw score [*r_s_*(170) = 0.299, *p* = 0.000], accounting for a small percentage of shared variance (8.94%).

### Working memory

In relation to working memory measures, the analysis revealed a non-significant positive trend between unusual experiences and letter–number sequencing raw score [*r_s_*(169) = 0.143, *p* = 0.064]. Moreover, exploratory analysis identified a positive non-significant trend between cognitive disorganization [*r_s_*(175) = 0.152, *p* = 0.051], impulsive non-conformity [*r_s_*(175) = 0.161, *p* = 0.038], and spatial span backwards raw score, which accounted for a small percentage of shared variance, 2.31 and 2.59%, respectively.

## Discussion

The current study aimed to explore associations between schizotypy factors and cognition in an adult community sample. The key findings from this study were significant positive associations between unusual experiences, cognitive disorganization, and impulsive non-conformity and inhibitory latency and cognitive flexibility on the Color–Word Interference Test, once baseline color naming and word reading were controlled. Additionally, findings revealed a significant positive association between introvertive anhedonia and attention and processing speed on the Trail Making Test – Part A. Lastly, results showed a significant negative association between introvertive anhedonia and reasoning and problem solving on the mazes task and a significant positive relationship between impulsive non-conformity and reasoning and problem solving on the mazes task.

Our findings of associations between positive, cognitive, and asocial schizotypal traits and impairments in inhibition and cognitive flexibility or attentional switching (Color–Word Interference Test) are in line with past studies that have found high schizotypes to display greater inhibitory latency and less accurate responses compared with low schizotypes on all inhibition and switching conditions of the Color–Word Interference Test (12). These findings are also in line with recent schizophrenia research, which has revealed inhibitory deficits using the Color–Word Interference Test (30). However, while the schizophrenia literature frequently reports a relationship with inhibitory deficits, negative symptoms are often related to inhibition, rather than positive symptoms (30). Albeit non-significant, results identified positive trends toward relationships between cognitive disorganization and impulsive non-conformity and cognitive flexibility, once baseline inhibition was controlled.

Furthermore, our results of a relationship between negative schizotypal traits and reduced attention and processing speed are consistent with past research reporting an association between negative and cognitive schizotypal traits and poorer sustained attention, as measured by the Continuous Performance Task, in a community sample (9). Similarly, these findings are in line with previous schizophrenia research that found patients to score significantly lower on the Continuous Performance Task compared with controls (31).

Additionally, the current study found a significant negative association between introvertive anhedonia and completion time on a mazes task, suggesting that higher levels of negative schizotypy are related to poorer reasoning and problem-solving performance. This is consistent with previous schizophrenia research that found patients to demonstrate significant impairment on a mazes task in comparison with controls (32). In contrast and somewhat counter intuitively, current results also revealed a significant positive association between impulsive non-conformity and superior reasoning and problem-solving performance. One potential explanation for this inconsistent finding is that faster task commencement times associated with impulsivity may have aided in participants’ increased mazes scores. In comparison with other mazes tasks, the current task did not penalize participant’s performance when they entered into a “blind alley.” For instance, the Porteous Maze task records a trial as unsuccessful if such behavior takes place (25). The current participants are likely to have benefited from the added speed associated with impulsivity without being punished for this commonly committed error.

### Limitations

A couple of noteworthy methodological shortcomings exist in the current study. For instance, schizotypy factor scores identified in this study were below current normative scores for the O-LIFE inventory (8). Due to the restricted range of schizotypy scores in the current sample, it is possible that relationships between schizotypy factor scores and neurocognitive variables may have been revealed using a sample with a larger spread of schizotypy scores.

In addition, previous literature has suggested the use of illicit drugs to impact both schizotypy scores and cognitive performance, particularly inhibition (33). Although the current study did exclude participants if they met criteria for a current substance disorder based on the M.I.N.I. screen, it did not, however, evaluate or control the current use of illicit drugs that were not severe enough to meet this criteria. As current use of cannabis can result in healthy individuals to mimic inhibitory impairments seen in schizophrenia, controlling the use of such substances may have been beneficial.

## Conclusion

In conclusion, the current study was one of the first in schizotypy literature to tease apart the relationships between factor scores and cognition in an adult community sample that accounted for psychiatric illness and family history. This allowed for the exploration of both cognitive functioning and potential compensatory mechanisms in individuals who have passed the peak onset times for developing schizophrenia. Findings from the current study further extend a limited body of schizotypy literature that enables inferences to be drawn in relation to the cognitive deficits seen in schizophrenia, without the potential confounds of illness chronicity and treatment medications. A better understanding of cognitive performance in schizophrenia is essential due to the vast experience of cognitive deficits and resistance to current treatment medications. Consequently, this research has potential practical implications for aiding in the establishment of treatments, to be used in conjunction with antipsychotic medication, for the cognitive symptoms of schizophrenia.

## Conflict of Interest Statement

The authors report no commercial or financial for the current research. Caroline Gurvich was funded by a National Health and Medical Research Council (NHMRC) early career fellowship.

## Supplementary Material

The Supplementary Material for this article can be found online at http://journal.frontiersin.org/article/10.3389/fpsyt.2015.00079/abstract

Table S1 **Spearman’s rho for neurocognitive variables by schizotypy factors**.
