# Supplementary material for: Schizotypal Traits are Associated with Poorer Executive Functioning in Healthy Adults
Source: Front Psychiatry. 2015 Jun 1;6:79. doi: 10.3389/fpsyt.2015.00079 (PMC4450583; doi:10.3389/fpsyt.2015.00079)
Supplement: Table S1 — Spearman’s rho for neurocognitive variables by schizotypy factors. [file Table_1.PDF]

## Supplementary Material

*Spearman's Rho for Neurocognitive Variables by Schizotypy Factors*

| | | D-KEFS Inhibition V Colour Naming | D-KEFS Inhibition/Switching V Inhibition | D-KEFS Inhibition/Switching V Colour Naming | D-KEFS Inhibition/Switching V Word Reading | Trail Making Test - Part A | Spatial Span Backwards | Letter-Number Sequencing | Mazes |
|---|---|---|---|---|---|---|---|---|---|
| Unusual Experiences | $r_s$ | .333** | .135 | .347** | .345** | .110 | .085 | .143 | .029 |
| | Sig. | .000 | .084 | .000 | .000 | .153 | .280 | .064 | .704 |
| | N | 164 | 166 | 166 | 166 | 169 | 165 | 169 | 169 |
| Cognitive Disorganisation | $r_s$ | .210** | .189 | .240** | .258** | .084 | .152 | .097 | .035 |
| | Sig. | .007 | .014 | .002 | .001 | .275 | .051 | .209 | .656 |
| | N | 164 | 166 | 166 | 166 | 169 | 165 | 169 | 169 |
| Introvertive Anhedonia | $r_s$ | -.039 | -.001 | -.048 | -.019 | .260** | -.059 | -.004 | -.212** |
| | Sig. | .619 | .994 | .543 | .806 | .001 | .454 | .962 | .006 |
| | N | 163 | 165 | 165 | 165 | 168 | 165 | 168 | 168 |
| Impulsive Nonconformity | $r_s$ | .450** | .168 | .453** | .433** | -.115 | .161 | .125 | .299** |
| | Sig. | .000 | .030 | .000 | .000 | .137 | .038 | .105 | .000 |
| | N | 165 | 167 | 167 | 167 | 170 | 166 | 170 | 170 |

*Note*. **p <.01 (2-tailed)
